# Clinical outcomes of active specific immunotherapy in advanced colorectal cancer and suspected minimal residual colorectal cancer: a meta-analysis and system review

**DOI:** 10.1186/1479-5876-9-17

**Published:** 2011-01-27

**Authors:** Benqiang Rao, Minyan Han, Lei Wang, Xiaoyan Gao, Jun Huang, Meijin Huang, Huanliang Liu, Jianping Wang

**Affiliations:** 1 Colorectal Surgery Department, The Sixth Affiliated Hospital, Sun Yat-sen University, Guangdong 510655, PR China; 2 Medical Department, The Sixth Affiliated Hospital, Sun Yat-sen University, Guangdong 510655,PR China; 3 Department of Pediatrics, The Sixth Affiliated Hospital, Sun Yat-sen University, Guangdong 510655, PR China; 4 Institute of Gastroenterology, Sun Yat-sen University, Guangzhou, Guangdong 510655, PR China

## Abstract

**Background:**

To evaluate the objective clinical outcomes of active specific immunotherapy (ASI) in advanced colorectal cancer (advanced CRC) and suspected minimal residual colorectal cancer (suspected minimal residual CRC).

**Methods:**

A search was conducted on Medline and Pub Med from January 1998 to January 2010 for original studies on ASI in colorectal cancer (CRC). All articles included in this study were assessed with the application of predetermined selection criteria and were divided into two groups: ASI in advanced CRC and ASI in suspected minimal residual CRC. For ASI in suspected minimal residual CRC, a meta-analysis was executed with results regarding the overall survival (OS) and disease-free survival (DFS). Regarding ASI in advanced colorectal cancer, a system review was performed with clinical outcomes.

**Results:**

1375 colorectal carcinoma patients with minimal residual disease have been enrolled in Meta-analysis. A significantly improved OS and DFS was noted for suspected minimal residual CRC patients utilizing ASI (For OS: HR = 0.76, P = 0.007; For DFS: HR = 0.76, P = 0.03). For ASI in stage II suspected minimal residual CRC, OS approached significance when compared with control (HR = 0.71, P = 0.09); however, the difference in DFS of ASI for the stage II suspected minimal residual CRC reached statistical significance (HR = 0.66, P = 0.02). For ASI in stage III suspected minimal residual CRC compared with control, The difference in both OS and DFS achieved statistical significance (For OS: HR = 0.76, P = 0.02; For DFS: HR = 0.81, P = 0.03). 656 advanced colorectal patients have been evaluated on ASI in advanced CRC. Eleven for CRs and PRs was reported, corresponding to an overall response rate of 1.68%. No serious adverse events have been observed in 2031 patients.

**Conclusions:**

It is unlikely that ASI will provide a standard complementary therapeutic approach for advanced CRC in the near future. However, the clinical responses to ASI in patients with suspected minimal residual CRC have been encouraging, and it has become clear that immunotherapy works best in situations of patients with suspected minimal residual CRC.

## Background

Colorectal cancer (CRC) is the third most common cancer in females and the fourth most common in males worldwide. CRC is the fourth and fifth most frequent cause of cancer-related deaths depending on gender [1]. Surgery is the cornerstone of CRC therapy. Unfortunately, more than 20% of patients with CRC have metastatic disease at the time of diagnosis [2]. Although the most common indication for liver resection in developed countries is metastatic CRC, surgery can only be performed in 20% patients [3].The prognosis of patients with resectable tumor depends on the disease stage. The 5-year survival for patients with CRC following surgery varies between 80-90% for stage I, 70-75% for stage II, 35-50% for stage III and < 7% for stage IV disease [4]. Despite the fact that 80% of CRC patients have complete macroscopic clearance of the tumor by surgery, 50% of CRC patients will relapse [5]. This is presumably due to the presence of micro-metastasis at the time of surgery. In general, the 5-year survival for patients with CRC ranges from 50-60% over the past 30 years [6].

Avenues for the clinical testing of rationally designed vaccination strategies, including immunotherapy, are being explored as complementary treatments. Recent advances in immunology and molecular biology have opened new fronts against cancer. Early strategies used for treatment of CRC included non-specific immunotherapies, such as exogenous immunostimulants, cytokines, adoptive transfer of non-specific immune effector cells, and the inhibition of negative immune regulatory pathways and tumor-derived immune suppressive molecules. Several studies have evaluated the clinical results to nonspecific immunotherapies in patients with CRC, but most of studies revealed no improvement in the response rate, progression-free survival, or overall survival [7-9]. In general, nonspecific approaches have yielded limited results in the treatment of CRC. Since the discovery of tumor-associated antigens during the early 1990s, rapid progress has been made in identifying antigens and describing immune interactions in cancer patients. Many clinical trials have been conducted using active specific immunotherapy (ASI) in CRC, including autologous tumor cell vaccines, define-tumor protein vaccines, monoclonal antibodies and anti-idiotype vaccines, multi- peptide vaccines, viral vector vaccine, DC vaccine, and naked DNA vaccine[10].

However, despite an abundance of preclinical data, relatively little is known regarding the efficacy of ASI in CRC. Early clinical trials of ASI against CRC have provided mixed results, which led to a controversy flare-up over the clinical efficacy of ASI in CRC [11,12]. In the present report, we focused on meta-analysis of ASI to patients with suspected minimal residual colorectal cancer (suspected minimal residual CRC), and reviewed the objective clinical outcomes of ASI in advanced colorectal cancer (advanced CRC) during the past 12 years.

## Methods

### Literature Search Strategy

A search was conducted on Medline and PubMed from January 1998 to January 2010 for original studies on ASI in CRC, Using the following keywords: "colorectal" OR "colon" OR "rectal" AND "cancer" OR "carcinoma" AND" vaccine "OR "vaccination" OR "immunization". Review papers were also examined for published results. We avoided duplications of data by examining the body of each publication and the names of all authors. When such duplications were identified, the latest version was included into our study.

### Selection Criteria

Inclusion criteria included all articles concerning histopathologically defined CRC treated by ASI. At the beginning of ASI, a minimum of 4-weeks should have elapsed from the time of completion of prior chemotherapy and/or radiation therapy. No concurrent chemotherapy, radiotherapy, or drugs which affect immune function (such as glucocorticoids, Cimetidine, etc.) should have been administered during ASI or follow-up. Studies were limited to human trials, and in the English language. Data regarding tumors without specific documentation of colorectal origin were not included. However, these exclusions were not applied if isolated data regarding CRC are provided. Case studies, review articles, and studies involving fewer than three patients were excluded to allow for consistent results.

### Data Extraction and Quality Assessment

Two reviewers independently selected the trials and performed the data extraction. Discrepancies were resolved by discussion among reviewers. Because the outcome parameters are different in advanced CRC and suspected minimal residual CRC, we divided the articles into two groups: ASI in advanced CRC (a measurable tumor burden) and ASI in suspected minimal residual CRC (patients had undergone complete resection for primary tumor or metastasis disease without evidence of remaining macroscopic disease). Clinical outcomes to evaluate ASI in suspected minimal residual CRC were OS and DFS, and clinical outcomes of ASI in advanced CRC were complete response (CR), partial response (PR), mixed or minor response (MR) and stable disease (SD), which had to meet the WHO criteria. To avoid ignoring small benefits that could add up to a clinically relevant result, the clinical benefit rate (CBR) has been introduced in this report. The CBR represents the sum of CR, PR, MR, and SD rates. Thus, for subset analysis, the CBR was calculated as the sum of CR, PR, MR, and SD based on the various vaccine formulations, the route of vaccination, and adjuvants [13].For the Meta-analysis of ASI in suspected minimal residual CRC, the overall quality of each study was assessed in accordance with the Jadad format[14]. A grading scheme (A, B, and C) is used to classify four main aspects: 1) quality of randomization, 2) quality of allocation concealment, 3) quality of blinding, and 4) quality of the description of withdrawals and dropouts. The grades are described as thus: A) adequate, with correct procedures, B) unclear, without a description of methods, and C) inadequate procedures, methods, or information. Based on these four criteria, the studies could be divided into three groups. "A" studies had a low risk of bias for studies and were scored with A grades for all items; "B" studies had a moderate risk of bias for studies with one or more B grades; "C" studies had a high risk of bias and were those with one or more C grades.

### Statistical Analysis

With regards to ASI in advanced CRC, a post hoc explorative analysis was performed to calculate the overall response rate of ASI as well as the clinical benefit rate, based on the various vaccine formulations, the route of vaccination, and adjuvants. For the ASI in suspected minimal residual CRC, statistical analysis was carried out using Review Manager (version 5.0) provided by The Cochrane Collaboration. Dichotomous data were presented as relative risk (HR) and continuous outcomes as weighted mean difference (WMD), both with 95% confidence intervals (CI). The overall effect was tested using Z scores, with significance being set at P < 0.05. Meta-analysis was performed using fixed-effect or random-effect methods, depending on absence or presence of significant heterogeneity [15]. Statistical heterogeneity between trials was evaluated by the chi-squared and I square (I^2^) tests, with significance being set at P < 0.10. In the absence of statistically significant heterogeneity, the fixed-effect method was used to combine the results. When heterogeneity was confirmed (P ≤ 0.10), the random-effect method was used.

## Results

### Quantity of Evidence

A total of 789 studies were identified by the searches. By scanning titles and abstracts, 548 redundant publications, reviews and case reports were excluded. After referring to full texts, 192 studies which did not satisfy the inclusion criteria were removed from consideration. A total of 49 studies were left for analysis which involved 2031 patients, of whom 1375 (6 studies) were included in ASI for suspected minimal residual CRC group, and 656 (43 studies) were included in ASI for advanced CRC group.

Table 1 shows the characteristics of the six trials included in the meta-analysis [16-21]. Three of the six trials reported data for 7 years follow-up, other three studies followed up for 1 year, 5 years and 7.6 years respectively. All six studies were randomized, three studies mentioned the concealment of allocation clearly in the randomization process, and two studies mentioned withdrawal rates; however, none of the trials was blinded. Accordingly, we considered two studies as category B, and four as category C.

**Table 1:** Clinical trials of ASI in suspected minimal residual CRC

Ref	ASI	Stage of patient	Overall Survival	Disease-free Survival	Follow up	Jadad's grades
					
			No. of events/no. of subjects		(year)	
[21]	ATC	Stage II	Con:31 of 109	Con:35 of 109	7.6	B
			Exp:16 of 73	Exp:18 of 73		
		Stage III	Con:26 of 44	Con:28 of 44		
			Exp:15of 33	Exp:15 of 33		
[22]	ATC-BCG	Stage II	Con:21 of 77	Con:29 of 77	5	C
			Exp:14 of 80	Exp:17 of 80		
		Stage III	Con:12 of 40	Con:17 of 40		
			Exp:16of 44	Exp:20 of 44		
[23]	ATV-NDV	Stage I-IV	Con:16 of 25	NO	7	C
			Exp:12of 25			
[24]	17-1	Stage III	Con:48 of 76	Con:54 of 76	7	B
	Antibody		Exp:39 of 90	Exp:50 of 90		
[25]	ATC	Stage I-IV	Con:146 of 257	NO	7	C
			Exp:135 of 310			
[26]	ATC	Stage IV	Con:48 of 50	NO	1	C
			Exp:20 of 42			

Abbreviations: Ref, reference; ASI, active specific immunotherapy; Con, control group; Exp, ASI experiment group; ATC, antilogous tumor cells; NDV, newcastle disease virus; No, not done.

Table 2 shows the characteristics of the 43 trials included in ASI for advanced CRC group [22-64]. Among 43 studies, all had clearly stated inclusion and exclusion criteria. In addition, all studies were described with comparable baseline characteristics of ASI, including the number of evaluated CRC patients, the type of vaccine, the route of vaccination, adjuvants, the toxicity, and the objective clinical responses.

**Table 2:** Clinical trials of ASI in advanced CRC

Ref	Vaccine	Adjuvant	Route	Patients	CR+PR	MR	SD
[27]	Anti-Id 3H1	i.c	AH	23	0	0	NR
[28]	CEA/HbsAg-CMV	i.m	HBsAg	17	0	0	0
[29]	DC-CEA peptid	i.v	No	10	1	1	2
[30]	ALVAC(CEA-B7.1)	i.m	ALVAC/B7.1	13	0	0	2
[31]	DC-CEA peptid	i.v	No	7	0	0	1
[32]	Auto-tumor	i.d	NDV	13	4	0	8
[33]	DC-CEA peptid	i.t	No	10	0	0	2
[34]	Virus CEA	s.c	GM-CSF\IL-2	11	0	0	NI
[35]	Virus CEA	i.d/s.c	Tricom/GM-CSF	35	0	0	14
[36]	DC-CEA peptid	s.c	No	7	0	0	2
[37]	SART3 peptide	s.c	IFA	12	0	0	1
[38]	DC-CEA transfected	i.v+id	IL-2	11	0	0	0
[39]	DC-CEA peptid	s.c+id	Tricom	11	0	0	6
[40]	DC + tumor RNA	i.v	KLH	15	0	0	0
[41]	DC+MAGE3 peptide	i.v	No	3	0	1	0
[42]	SART-IcK-CyB multi peptide	s.c	Montanideisa-51	10	0	1	1
[43]	Survive peptide	s.c	No	17	0	1	3
[44]	DC + CEA peptide	s.c/i.d	No	11	0	0	3
[45]	ALVAC expressing CEA+B7.1	i.d	B7.1	28	0	0	7
[46]	Autologous hemoderivative	s.c	GM-CSF	50	0	0	26
[47]	DC+allogeneic tumor cell lysate	i.d	No	17	0	0	4
[48]	TroVax	i.m	MVA	17	0	0	5
[49]	P53-SLP	s.c	No	10	0	0	4
[50]	tumor lysate pulsed-Dc	i.t	THI	8	0	0	4
[51]	Aex+GM-CSF	s.c	GM-CSF	20	0	1	1
[52]	DC+MHC-I peptide	i.d	IFN-[r]/GM-CSF	11	0	0	0
[53]	Glutaraldehyde-fixed HUVECs	i.d	No	3	0	0	0
[54]	Xenogenic polyantigenic vaccine	s.c	IL-2	37	2	10	11
[55]	Oncolytic poxvirus JX-594	PEIT	GM-CSF	4	0	0	3
[56]	MIDGE	s.c	d-SLIM	10	2	1	2
[57]	ALVAC-p53	i.v	ALVAC	16	0	0	1
[58]	ONYX-015 adevirus	i.v	No	18	0	0	7
[59]	TNFa AutoVaccIne	i.m	AH	33	2	0	7
[60]	rF-CEA-TRICOM	i.d	B7.1	11	0	1	4
[61]	CEA alt-plused DC	iv	No	7	0	0	1
[62]	DC-CEA peptid	i.t	IL-4/GM-CSF	10	0	0	2
[63]	Murine monoclonal CEA-antibody	i.d	AH	15	0	0	1
[64]	Ep-CAM protein	s.c	MPL/GM-CSF	11	0	0	3
[65]	Vaccine virus expressing CEA	i.d/s.c	No	20	0	0	2
[66]	DC + CEA peptide	i.v/i.d	IL-2	11	0	0	0
[67]	Antibody SCV 106 mimicking 17-1A	s.c	AH	21	0	0	0
[68]	Autologous tumor	s.c	Fibroblasts/IL-2	10	0	0	1
[69]	retroviral vector- IL-2 allogeneic tumor cells + IL-1a	i.d	DETOX/IL-1a	22	0	2	0

**Total**	**43**			**656**	**11(1.68%)**	**19(2.9%)**	**141(21.49%)**

Abbreviations: Ref, reference□AH: aluminum hydroxide; NI, not identifiable; NR, not Reported; DC, dendritic cells□NDV, newcastle disease virus; IL, interleukin; ß-HCG, ß-human chorionic gonadotropin; THI,tetanustoxoidntigen/hepatitis B/influence matrix peptide; IFA, incompleteFreund's adjuvant.

### Meta-analysis of ASI in suspected minimal residual CRC

**Figure 1:**
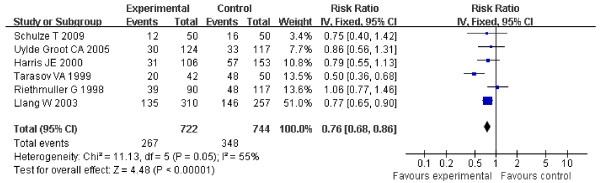
**Forest plot of comparison: Overall Survival of 6 included study (stage I-IV)**.

The OS at the end of treatment for ASI in patients with suspected minimal residual CRC is shown in Table 1. For stage I-IV suspected minimal residual CRC, statistically significant heterogeneity was detected (Tau2 = 0.03, Chi2 = 11.13, df = 5, P = 0.05, I2 = 55%) (Figure 1), using the random-effect method for meta-analysis. HR for ASI in stage I-IV suspected minimal residual CRC was 0.76 (95% CI 0.63-0.93), the difference of OS at the end of follow-up between the ASI in stage I-IV suspected minimal residual CRC group and control groups was statistically significant (Z = 2.68, P = 0.007) (Figure 1).

**Figure 2:**
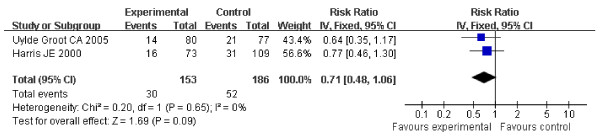
**Forest plot of comparison: Overall Survival of stage II (2 study)**.

**Figure 3:**
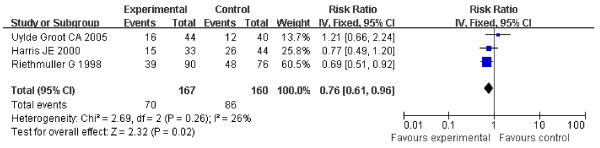
**Forest plot of comparison: Overall Survival of stage III**.

For stage II or III suspected minimal residual CRC, There were no statistical heterogeneity (Heterogeneity for stage II: Chi2 = 0.20, df = 1, P = 0.65, I2 = 0%; for stage III: Chi2 = 2.69, df = 2, P = 0.26, I2 = 26%) allowing the use of a fixed effect model for meta-analysis (Figure 2, 3). HR for stage II was 0.71 (95% CI 0.48-1.06, Z = 1.69, P = 0.09) (Figure 2), and HR for stage III was 0.76 (95% CI 0.61-0.96, Z = 2.32, P = 0.02) (Figure 3). For ASI in stage II suspected minimal residual CRC, OS approached significance (P = 0.09) when compared with control; however, the difference in OS of ASI for the stage III suspected minimal residual CRC reached statistical significance.

**Figure 4:**
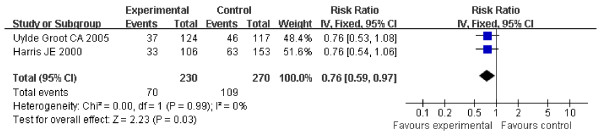
**Forest plot of comparison: Disease-free Survival of 3 study (Stage II and stage III)**.

**Figure 5:**
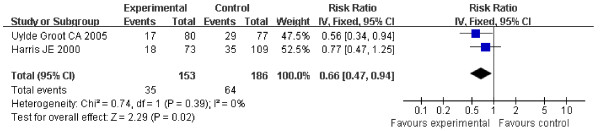
**Forest plot of comparison: Disease-free Survival of stage II**.

**Figure 6:**
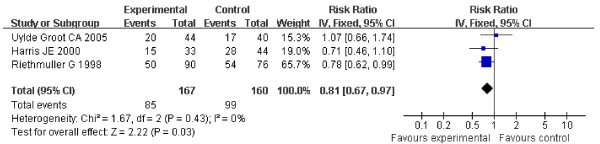
**Forest plot of comparison: Disease-free Survival of stage III**.

The DFS of the patients in three studies at the end follow-up is shown in table 1. These included 666 patients and showed the HR for DFS in stage II and stage III suspected minimal residual CRC was 0.76 (95% CI 0.59-0.97, Z = 2.23, P = 0.03) (Figure 4), which showed ASI in stage II and stage III suspected minimal residual CRC was markedly effective in term of DFS. No statistical heterogeneity was found (Chi2=0.00, df=1, P=0.99, I2=0%) (Heterogeneity for stage II-III suspected minimal residual CRC: Chi2 = 0.00, df = 1, P = 0.99, I2 = 0%; for stage II Chi2 = 0.74, df = 1, P = 0.39, I2 = 0%; for stage III: Chi2 = 1.67, df=2, P = 0.43, I2 = 0%) (Figure 4, 5, 6), allowing the use of a fixed effect model for meta-analysis. The HR for DFS in stage II suspected minimal residual CRC was 0.66 (95% CI 0.47-0.94, Z = 2.29, P = 0.02) (Figure 5), compared to a 0.81 HR in stage III suspected minimal residual CRC (95% CI 0.67-0.97, Z = 2.22, P = 0.03) (Figure 6). The results revealed that ASI in stage II suspected minimal residual CRC was more effective than in stage III suspected minimal residual CRC in term of DFS.

### Assessment of ASI in advanced CRC

For analysis of ASI in advanced CRC, 656 patients were evaluated for clinical responses. Eleven patients reported CR and seventeen reported PR, out of a total population of 656 patients, which corresponded to an overall response rate of 1.68%. MR was reported in 2.90% of patients; SD was found in 21.49%. The combined percentages of CR, PR, MR, and SD for all patients yielded a CBR of 26.07% (Table 2).

In 43 studies of ASI in advance CRC, patients received a variety of vaccinations including dendritic cells in fourteen studies, viral vector vaccines in ten, peptide in eight, autologous or allogeneic tumor cells or tumor-derived products in five, monoclonal antibodies and anti-idiotype vaccines in four, and other substances in five studies (naked DNA vaccine, define-tumor protein vaccine, autologous hemoderivative cyclophosphamide, glutaraldehyde-fixed HUVECs and xenogenic polyantigenic vaccine). CBR of 45/142 (31.7%) for multi-peptide vaccines, 17/70 (28.6%) for autologous tumor cell vaccine, 46/163 (28.2%) for viral vector vaccine, 30/134 (22.4%) for dendritic cell-based vaccines (Table 3).

**Table 3:** Clinical benefit rate of ASI with diffident type of vaccines in advanced CRC, post hoc explorative analysis

vaccine	clinical benefit rate
autologous tumor cell	20/70(28.6%)

Peptide vaccine	45/142(31.7%)

Viral vector vaccine	46/163(28.2%)

DC vaccine	30/134(22.4%)

Despite the broad variety of antigens described, carcinoembryonic antigen-based vaccination was used in 18 studies included in the present review. 1 PR, 2 MR, and 49 SD were reported in a total population of 256 patients (CBR = 20.3%). Fifteen further substances were used as adjuvants, Ten studies were done without adjuvants. Vaccines were administrated by different routes of injection: s.c. in ten studies, i.d. eight studies, i.m. five studies, i.v. four studies, i.d. and s.c. five studies, i.v. and i.d. three studies, and intralymphatic/intranodal two studies. In a post hoc analysis, The CBR ranged between 19.7% and 34% regardless of the route of vaccination (Table 4).

**Table 4:** Influence of vaccination route, post hoc explorative analysis

vaccine	clinical benefit rate
**s.c**	73/215(34.0%)
**i.m**	16/80(20.0%)
**i.d**	31/120(25.0%)
**i.v**	15/76(19.7%)

### Assessment of Toxicity for ASI in CRC

The current clinical experience with ASI does not indicate considerable toxicity. Neither short-term serious adverse events nor long-term autoimmune side effects have been observed using therapeutic vaccines in a large number of patients. The most frequently reported adverse events causally related to the use of ASI were mild (grade 1-2) in severity, including injection site reactions (e.g, erythema, pruritus, pain), fever, nausea, and fatigue. There were no significant hepatic, renal, pulmonary, cardiac, hematologic, or neurologic toxicities attributable to the treatments. No clinical manifestations of autoimmune reactions were observed. No significant changes in temperature and blood pressure were recorded. Other side effects include rare cases of adenopathy, diarrhea, rigors, malaise, and transfusion-like reactions. All other symptoms were described only in single cases and/or are most probably due to the advanced malignant disease or a side effect of adjuvants.

## Discussion

According to our Meta-analysis, all patients with suspected minimal residual CRC who met quality control specifications and protocol eligibility (analyzable patients), OS (P = 0.007), and DFS (P = 0.003) were significantly improved when compared with controls. A subgroup analysis by stage of disease, For ASI in stage II suspected minimal residual CRC compared with control, OS approached significance when compared with control (P = 0.09), The DFS of ASI reached statistical significance (P = 0.02); For ASI in stage III suspected minimal residual CRC compared with control, The difference in both OS (P = 0.02) and DFS (P = 0.03) achieved statistical significance. These results indicated ASI may provide a new promising targeted therapeutic approach in suspected minimal residual CRC.

The efficacy of ASI in patients with suspected minimal residual CRC is encouraging and merit generalization in colorectal cancer therapy based on three reasons. First, in less than a decade, because of improved diagnostic methods, there has been a major shift from stage IV to stage II CRC. In 1995, stage IV disease accounted for approximately 50% to 55% of all cases, stage III accounted for 30%, and stage II for less than 20%. For the year 2004, it is estimated that stage IV cancers will account for approximately 10% of all cases, while stage II disease will rise to 40% of all cases [65]. This progression is expected to continue through the rest of the decade, which means more and more CRC patients would procure benefits with ASI. Second, micro metastases are generally responsible for disease recurrence and the eventual death of CRC patients. Occult micro metastases or suspected minimal residual CRC have been detected in lymph nodes or in the operating field in 54% of stage II patients. Analysis of the relationship between PCR-detectable metastases and survival has resulted in an adjusted five year survival of 91% in patients without minimal residual CRC and 50% in patients with minimal residual CRC, with observed five year survival rates of 75% and 36%, respectively [66]. Hence, the development of new methods of treatment to eliminate micro metastases in patients with suspected minimal residual CRC and thereby delay or prevent recurrence is particularly urgent given the increasing incidence of CRC. Third, cancer stem cells may be responsible for tumor recurrence and metastatic lesions, and have been postulated to be a very small population of quiescent or very slowly dividing cells within a growing tumor mass. Such cells would be inherently resistant to treatments such as chemotherapy, which target proliferating cells [67]. Since the proliferation is not a prerequisite for recognition and destruction by the immune mechanisms, ASI may be the most effective way to eliminate cancer stem cells, ASI is likely to be applied in the setting of curatively minimal residual cancer with the goal of clearing the invisible but present cancer burden.

The efficacy of ASI in patients with advanced CRC was disappointed. Nagorsen *et al *evaluated the outcomes of ASI in advanced CRC from January 1985 to January 2006, which revealed a very weak clinical response rate of 0.9% for ASI procedures available for advanced CRC [13]. In the present system review, we found an objective response rate of 1.68% over 656 advanced CRC patients treated with ASI in 43 different studies. Peptide vaccination had the highest CBR of 31.7%, followed by 28.6% for autologous tumor vaccines, 28.2% for viral vector vaccine, and 24.4% for DC-based therapy. These data are two-fold higher than those reported by Nagorsen *et al*. Our study has demonstrated that ASI in CRC has made recent progression.

However, although progression was conspicuous with ASI in advanced CRC, the clinical results are still limited. As new generations of vaccines are developed to improve the clinical efficiency, several considerations will require attention. First, because chemotherapy is standard in the treatment of CRC, it is important to demonstrate whether immunizations may be given to patients who are receiving systemic chemotherapy. This opportunity rests in strategically combining immunotherapies with both traditional and novel cancer drugs to shape both the global host environment and the local tumor environment, and to ameliorate distinct layers of immune tolerance, ultimately supporting a vigorous and sustained antitumor immune response [68]. Within this modified host environment, ASI regimens that (1) combine tumor vaccines or tumor-specific lymphocytes with targeted drugs that amplify the magnitude and quality of end immune effectors and (2) relieve the normal controls at specific points in the process of T cell activation will be critical for success [69]. More importantly, chemotherapeutic drugs kill tumor cells and, in the process, increase the amount of tumor antigens that are presented to immune system. Moreover, the process of apoptotic cell death may in itself provide an immunostimulatory signal. Both have the capacity to enhance antitumor immune responses. Second, ASI effectiveness depends on tumor burden. An advanced cancer actually induces Tregs and then uses them to subvert the immune response of ASI [70]. The implication is that the Tregs contribute to the inability of immune system to eliminate the growing tumor. It is thus apparent that effective ASI should include approaches that target Tregs in vivo. Several strategies have been employed with certain efficacy in cancer, including depletion with anti-CD25 antibodies, treatment with anti-GITR and anti-CTLA-4 [71-73]. The findings suggest depletion Tregs may be used in the future to improve immunotherapy in CRC [74]. Third, it may be more important to choose antigens that have functions important to the cancer cell. Some researchers have argued that immunologically targeting proteins without a known protumorigenic function may ultimately fail because tumors could down-regulate these antigens without a detrimental effect to their function [75]. As new generations of vaccines are developed, DNA vaccination is a promising avenue for the development of a successful CRC vaccine [76]. However, there is only one clinical trial which utilizes a DNA vaccine for CRC [22]. We agree with those who find it premature to give up on active cancer vaccines, although much work remains.

## Conclusions

In summary, This Meta-analysis and System Review clearly supports the idea that a statistically significantly improved DFS or OS was shown in all stage suspected minimal residual CRC patients. Meanwhile, there was also a clear indication that the objective clinical outcome of ASI in advanced CRC was only 1.6%. The results showed it is unlikely that ASI will provide a standard complementary therapeutic approach for advanced CRC in the near future. However, it has become clear that immunotherapy works best in situations of patients with suspected minimal residual CRC.

## Competing interests

The authors declare that they have no competing interests.

## Authors' contributions

JW conceived the study, provided funding support, and revised the manuscript critically for important intellectual content. BR made substantial contributions to the design, acquisition, analysis, and interpretation of data. MH, LW, MH, XG, HL and JH participated in the design, acquisition, analysis and interpretation of data. All authors approved the final manuscript.
